# Progress in Mozambique: Changes in the availability, use, and quality of emergency obstetric and newborn care between 2007 and 2012

**DOI:** 10.1371/journal.pone.0199883

**Published:** 2018-07-18

**Authors:** Orvalho Augusto, Emily E. Keyes, Tavares Madede, Fátima Abacassamo, Pilar de la Corte, Baltazar Chilundo, Patricia E. Bailey

**Affiliations:** 1 Universidade Eduardo Mondlane, Faculdade de Medicina, Maputo, Moçambique; 2 FHI 360, Durham, North Carolina, United States of America; 3 Averting Maternal Death & Disability, Columbia University, New York, NY, United States of America; 4 UNFPA, Maputo, Mozambique; University of Heidelberg, GERMANY

## Abstract

**Introduction:**

Maternal mortality in Mozambique has not declined significantly in the last 10–15 years, plateauing around 480 maternal deaths per 100,000 live births. Good quality antenatal care and routine and emergency intrapartum care are critical to reducing preventable maternal and newborn deaths.

**Materials and methods:**

We compare the findings from two national cross-sectional facility-based assessments conducted in 2007 and 2012. Both were designed to measure the availability, use and quality of emergency obstetric and neonatal care. Indicators for monitoring emergency obstetric care were used as were descriptive statistics.

**Results:**

The availability of facilities providing the full range of obstetric life-saving procedures (signal functions) decreased. However, an expansion in the provision of individual signal functions was highly visible in health centers and health posts, but in hospitals, performance was less satisfactory, with proportionally fewer hospitals providing assisted vaginal delivery, obstetric surgery and blood transfusions. All other key indicators showed signs of improvements: the institutional delivery rate, the cesarean delivery rate, met need for emergency obstetric care (EmOC), institutional stillbirth and early neonatal death rates, and cause-specific case fatality rates (CFRs). CFRs for most major obstetric complications declined between 17% and 69%. The contribution of direct causes to maternal deaths decreased while the proportion of indirect causes doubled during the five-year interval.

**Conclusions:**

The indicator of EmOC service availability, often used for planning and developing EmONC networks, requires close examination. The standard definition can mask programmatic weaknesses and thus, fails to inform decision makers of what to target. In this case, the decline in the use of assisted vaginal delivery explained much of the difference in this indicator between the two surveys, as did faltering hospital performance.

Despite this backsliding, many signs of improvement were also observed in this 5-year period, but indicator levels continue below recommended thresholds. The quality of intrapartum care and the adverse consequences from infectious diseases during pregnancy point to priority areas for programmatic improvement.

## Introduction

Mozambique’s maternal mortality ratio continues to be high at 480/100,000 live births after a decrease of 4.2% yearly during the last two decades [1–3]. The neonatal mortality rate, also high, was last estimated at 30/1000 live births [1]. Neither Millennium Development Goal 4 nor 5 was achieved by 2015.

Nationally representative indicators from two censuses in 1997 and 2007, three Demographic and Health Surveys (DHS) in 1997, 2003 and 2011, and one Multiple Indicator Cluster Survey (MICS) in 2008 have allowed the tracking at the population level of service coverage, service utilization and maternal and child health (MCH) outcomes. Routine data from the health information system have been used to follow the deployment, implementation and administration of MCH services. Program planning and policy setting benefit from both population surveys and their complement of health facility surveys that provide key information on the capacity of the health system to deliver high impact maternal and newborn interventions.

To fill this supply-side need, many low- and middle-income countries have conducted EmONC assessments [4, 5]. These assessments collect information on the availability, use and quality of routine and emergency obstetric and newborn care (EmONC). They offer a standardized status assessment of health facilities with sufficient details to identify gaps, set policies and prioritize interventions for implementation. They also provide flexibility through the adaptation of tools to capture country specific health system elements [6].

Two standard features of these assessments include: 1) monitoring the performance of the nine emergency obstetric care (EmOC) signal functions–a set of life-saving interventions that treat the major direct causes of maternal mortality, and 2) the calculation of eight EmOC indicators, which have been widely used over two decades or more [6–9]. Often the focus has been on the first indicator–the availability of emergency obstetric and newborn care–and has included the use of geospatial techniques to illustrate the importance of distance or travel time in determining access [10–14]. The utilization indicators cover the institutional delivery rate, the population-based cesarean rate and met need for EmOC, the subject of a recent systematic review to determine the global met need for EmOC (the proportion of women who are expected to develop severe complications in pregnancy or childbirth who receive treatment) [15]. Two indicators function as proxies for quality of care: an aggregate of the case fatality rate and the intrapartum stillbirth and early neonatal death rate. Lastly, the proportion of maternal deaths due to indirect causes flags the need for maternal services in addition to EmOC (6).

Although one of the stated objectives of an EmONC assessment is to collect baseline estimates, few countries have taken advantage of more than one national assessment to show change in these indicators [16]. In this article, we investigate changes in the availability, use and quality of EmONC services between 2007 and 2012 in Mozambique. To fully understand changes in availability, we examine specific signal functions by type of facility and volume of deliveries. By comparing the results of two EmONC assessments we can assess where progress has been achieved and where gaps remain.

## Materials and methods

### Study design

We conducted secondary data analysis based on two nationally representative cross-sectional EmONC assessments that targeted health facilities providing childbirth services.

### Study setting

Mozambique is a southern East African country, with 801,590 km^2^ of surface. The World Bank classifies Mozambique as a low-income country; the gross national income per capita (average annual income) in 2015 was US$590 [17]. According to the UNDP 2014 Human Development Index, Mozambique was ranked 180 [18], an improvement over its ranking just a few years ago.

Administratively, the country is divided into 11 provinces and the capital city of Maputo. Important rivers cross the country from west to east, isolating parts of the country during the rainy season. Currently it has 26.4 million inhabitants with half of the population under the age of 18 [19]. The country is among the eight most HIV prevalent countries in the world with 11.5% of its adult population infected [20].

The national health system (NHS) provides 90% of all health care services throughout the country, while private sector facilities are available primarily in Maputo City. The NHS is divided into four levels of progressively more complex care. The first level provides primary care (health centers or posts), where most facilities provide basic maternal and child health services; the secondary level functions at the district level and as a referral level for the first, and may offer surgical services including cesareans (rural, district or general hospitals); the tertiary level (provincial hospitals) functions as a referral level located in provincial capitals, and the quaternary level (central hospitals) serves as the regional referral level. This last level falls directly under the central government budget while the other three levels are impacted by budgeting at central level as well as local government.

In answer to a lack of obstetric specialists, assistant medical officers (surgical technicians) are trained to perform major obstetric surgery, especially in rural areas [21]. To provide routine and basic emergency obstetric care (B-EmOC) three levels of nurses exist: basic and mid-level maternal-child health nurses and higher level nurses, or maternal health nurses, with 1.5, 2.5 and 5 years of training, respectively, in maternal and child health.

### Data sources

The assessments collected a broad range of facility specific information: the availability of infrastructure, equipment, consumable supplies, drugs, human resources, service statistics, and the performance of key life-saving procedures, routine delivery and newborn services. Service statistics were extracted from facility registers including the number of deliveries (by mode of delivery), obstetric complications (by type), maternal deaths (by cause), and newborn outcomes. For this paper, we relied primarily on the service statistics and interviews with facility staff on the performance of essential services including the nine EmOC signal functions. A complete discussion of assessment methodologies can be found in the assessment reports [22, 23].

Health facilities were identified through the national health information system and were included in the sampling frame if at least one delivery had been reported to the health information system in the most recent 12-month period. Government-managed facilities dominate the health system in Mozambique; in 2007, fewer than 3% of facilities were private or managed by a private-public partnership. The 2007 EmONC assessment sample consisted of 427 facilities: this included all hospitals, health centers from district capitals and type I health centers and a 40% sample of lower level facilities (type II and III health centers and health posts), stratified by province and type of facility. The data was weighted to represent the 877 health facilities on the sampling frame. Data collection occurred between November 2007 and January 2008 and covered service statistics from the 12-month period of November 2006 through October 2007.

The 2012 EmONC assessment was a census of health facilities that offered childbirth services and included 946 facilities; a small proportion (7% or 66) were private not-for profit facilities, most of which were under private-public management. Data collection occurred between November and December 2012, but unlike the 2007 assessment the reference period for the retrospective service statistics covered the three months before the assessment, from 1 August to 31 October 2012.

To estimate the number of expected births, we used the 2007 population of 20,530,715 and a crude birth rate of 45/1000 live births based on the 2007 Census [20]. The 2012 population estimate was 23,700,000 with a crude birth rate of 41.1 based on the 2012 Statistical Yearbook for Mozambique [24]. Since the writing of the 2012 assessment report, the crude birth rate for that year was revised upward by the National Institute for Statistics, thus causing some deviation from the original results found in the 2012 assessment report.

### Definitions

A health facility was considered a B-EmOC facility if it performed the seven B-EmOC signal functions in the three months prior to the survey (parenteral antibiotics, uterotonics, anticonvulsants, manual removal of placenta, removal of retained products, assisted vaginal delivery, and neonatal resuscitation). A comprehensive EmOC (C-EmOC) facility must have performed all nine signal functions in the three months prior to the survey (the seven basic signal functions plus cesarean delivery and blood transfusion). When facilities performed all the signal functions (basic or comprehensive), we considered them “fully functioning.” The global recommendation is at minimum five fully functioning EmOC facilities–with at least one functioning at the comprehensive level–per 500,000 inhabitants or 20,000 births. Definitions of the EmOC indicators and signal functions, as well as the UN recommendations, are described further in *Monitoring emergency obstetric care*: *A handbook* (6). Note that the assessments are referred to as EmONC assessments because they contain substantial information on newborn care, but the signal functions and indicators are referred to as EmOC since they do not adequately cover the full range of interventions that small and sick newborns require. Indicators and signal functions for the newborn have been proposed elsewhere and are currently under discussion at the global level [25].

### Research ethics

Prior to data collection in 2007, the research team sought and received approval from the National Committee for Bioethics in Health (IRB 00002657), Ministry of Health in Mozambique [20]. In 2012, the team turned to the Department of Reproductive, Child and Adolescent Health, within the Mozambique Ministry of Health’s National Directorate for the Promotion of Health and Disease Control [23]. In both surveys, no names or identifying characteristics of patients were collected. No further approval was sought for the writing of this manuscript since it reflects secondary data analysis of the two original surveys.

### Data processing and statistical procedures

The 2007 data were entered using CSPro version 3.2 and analyzed using Stata. The data entry and management process was challenged by limited resources (human and time) and some facility data were compromised, sometimes reducing the usable dataset from 427 to 378 facilities. However, attempts to correct the loss of data were made through additional weighting steps. Thus, the 2007 data were weighted based on the probability of selection, but also by module, when these were missing. Data processing for 2007 has been described elsewhere [22]. For 2012, data were entered in EpiData 3.1 and exported to Stata (Release 13) for analysis [26, 27]. Descriptive statistics (frequencies, percentages, means, medians and ranges) were employed.

## Results

### EmOC indicators–measuring availability at national and provincial levels

In 2007, the national health system had 38% of the targeted or minimum recommended EmOC facilities and 79% of the recommended comprehensive facilities (Table 1). In 2012, the availability of EmOC had decreased to 28% of the recommended number of EmOC facilities and 67% of the recommended number of C-EmOC facilities. The absolute number of fully functioning B-EmOC facilities dropped by 11 and the number of fully functioning C-EmOC facilities dropped by one, but given the increase in the population, the availability of fully functioning B-EmOC and C-EmOC facilities for the population deteriorated.

**Table 1:** Availability of EmOC facilities (EmOC indicators 1 and 2) in 2007 and 2012, by region.

	2007							2012						
	Population^1^	B-EmOC and C-EmOC facilities			C-EmOC facilities			Population^1^	B-EmOC and C-EmOC facilities			C-EmOC facilities		
		Target^2^	How many exist	Existing as percent of target	Target^2^	How many exist	Existing as percent of target		Target^2^	How many exist	Existing as percent of target	Target^2^	How many exist	Existing as percent of target
		n	n	%	n	n	%		n	n	%	n	n	%
**National**	20,539,715	206	78	38%	42	33	79%	23,700,715	238	67	28%	48	32	67%
**Province**														
Niassa	1,178,117	12	9	75%	3	2	67%	1,472,387	15	7	47%	3	3	100%
Cabo Delgado	1,632,809	17	4	24%	4	3	75%	1,797,335	18	7	39%	4	4	100%
Nampula	4,076,642	41	14	34%	9	4	44%	4,647,841	47	6	13%	10	4	40%
Zambezia	3,892,854	39	9	23%	8	2	25%	4,444,204	45	7	16%	9	3	33%
Tete	1,832,339	19	4	21%	4	3	75%	2,228,527	23	7	30%	5	3	60%
Manica	1,418,927	15	6	40%	3	1	33%	1,735,351	18	8	44%	4	2	50%
Sofala	1,654,163	17	12	71%	4	7	175%	1,903,728	20	7	35%	4	4	100%
Inhambane	1,267,035	13	10	77%	3	3	100%	1,426,684	15	8	53%	3	3	100%
Gaza	1,219,013	13	7	54%	3	5	167%	1,344,095	14	6	43%	3	3	100%
Maputo	1,259,713	13	0	0%	3	0	0%	1,506,442	16	1	6%	4	0	0%
Maputo City	1,099,193	11	3	27%	3	3	100%	1,194,121	12	3	25%	3	3	100%

^1^ Source: reference 20 for 2007 and 22 for 2012.

^2^ Target or recommendation is a minimum of 5 EmOC (combination of B-EmOC and C-EmOC) facilities per 500,000 population, where at least 1 is comprehensive. Source: WHO, UNFPA, UNICEF, AMDD. Monitoring emergency obstetric care: a handbook. Geneva: World Health Organization, 2009.

Table 1 also shows the availability of fully functioning EmOC facilities at the provincial level. Six provinces achieved less of the minimum target in 2012 compared to 2007, four showed improved EmOC availability, and one province (Maputo City) remained about the same. Two provinces showed steep backsliding in terms of meeting the recommended number of EmOC facilities: Nampula dropped from 34% to 13%, a 62% reduction, and Sofala went from 71% to 35%, a 51% reduction.

To better understand the decreased availability of EmOC facilities, we examined the performance of individual signal functions by level of care to identify which were missing in 2012 (Table 2). The percentage of hospitals that conducted each signal function marginally increased or remained the same except for assisted vaginal delivery (AVD), cesarean delivery, and blood transfusion. The single most dramatic drop among the signal functions at hospitals, however, was AVD, which decreased by 14 percentage points, from 89 to 75%. Health centers and health posts, on the other hand, showed more favorable overall results than hospitals. In 2012, substantial increases were observed for five of the seven basic signal functions, while the level of parenteral antibiotics performance remained the same and AVD decreased by half, from 23% to only 11%. In 2007 16% of health centers reported the provision of blood transfusion and the same percentage reported obstetric surgery, but by 2012, no health center or post reported performing cesarean delivery, and only 10% had provided a blood transfusion in the three months before the visit to the facility.

**Table 2:** Percent of facilities that performed each signal function in the 3-months before the survey, by facility type and year.

	Hospitals		Health Centers/Posts	
Signal functions	2007^a^	2012	2007 ^a^	2012
	(N = 41)	(N = 55)	(N = 337)	(N = 891)
Parenteral antibiotics	97.8%	100.0%	55.9%	57.0%
Parenteral oxytocics	100.0%	100.0%	76.6%	94.9%
Parenteral anticonvulsants	88.9%	94.5%	25.8%	45.6%
Manual removal of placenta	91.3%	94.5%	32.6%	53.9%
Removal of retained products	95.6%	94.5%	42.4%	60.9%
Assisted vaginal delivery	89.1%	74.5%	22.9%	11.0%
Neonatal resuscitation	97.8%	100.0%	42.6%	68.0%
Blood transfusion	93.5%	89.0%	16.2%	10.0%
Cesarean delivery	86.7%	78.2%	15.7%	0

^a^For 2007, the N’s are unweighted but the percentages are weighted.

**Fig 1:**
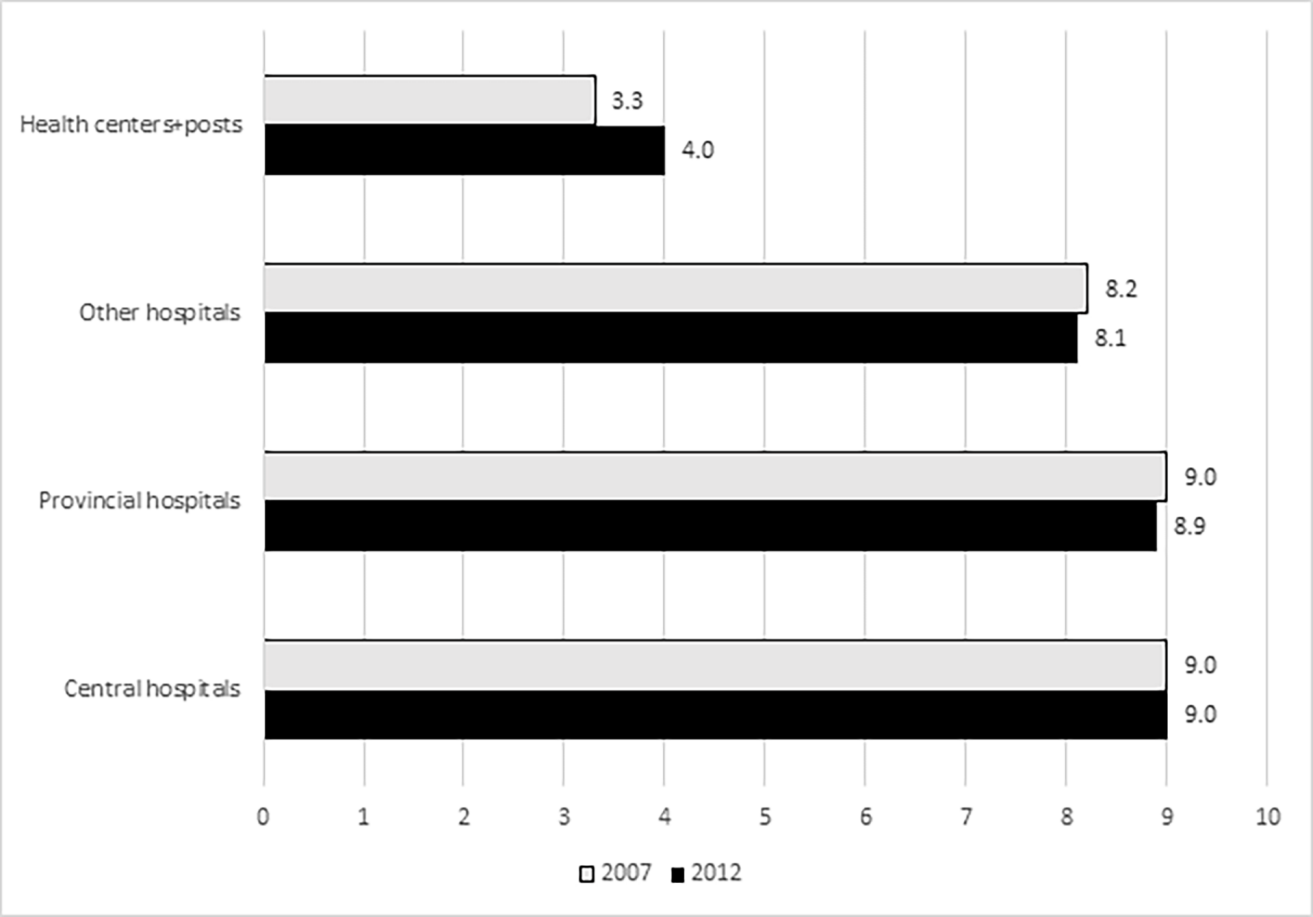
Comparison of the mean number of signal functions performed in the 3 months prior to the survey, by type of facility and year.

We next looked at how many signal functions facilities were performed in the previous three months by facility type (Table 3). No difference in performance was noted among the three central hospitals but one provincial hospital in 2012 had performed only eight signal functions. Among the other hospitals–the general, rural and district hospitals–in 2007, almost two-thirds had performed all nine of the signal functions while in 2012 only 51% had done so. The major difference between the two surveys was the increased reporting of signal function performance among health centers and posts in 2012. In 2007 15% had reported no signal functions compared to 2% in 2012. The mean number of signal functions at this level increased by 21 percent, from a mean of 3.3 signal functions in 2007 to 4.0 in 2012, despite the reduction in the use of AVD (Fig 1).

**Table 3:** Percent distribution of the number of signal functions performed, by type of facility and year.

	Central Hospitals		Provincial Hospitals		Other Hospitals		Health centers & posts	
Number of signal functions performed in last 3 months	2007 N = 3	2012 N = 3	2007 N = 7	2012 N = 7	2007 N = 31	2012 N = 45	2007 N = 337	2012 N = 891
0	0.0	0.0	0.0	0.0	0.0	0.0	15.3	1.8
1	0.0	0.0	0.0	0.0	0.0	0.0	15.5	7.3
2	0.0	0.0	0.0	0.0	2.9	0.0	12.7	13.1
3	0.0	0.0	0.0	0.0	0.0	0.0	11.7	16.9
4	0.0	0.0	0.0	0.0	0.0	0.0	9.7	19.4
5	0.0	0.0	0.0	0.0	2.9	4.4	14.4	18.7
6	0.0	0.0	0.0	0.0	5.7	8.9	8.5	15.0
7	0.0	0.0	0.0	0.0	5.7	8.9	4.9	5.9
8	0.0	0.0	0.0	14.3	17.1	26.7	6.1	1.7
9	100.0	100.0	100.0	85.7	65.7	51.1	0.0	0.0
Mean	9.0	9.0	9.0	8.9	8.2	8.1	3.3	4.0

**Fig 2:**
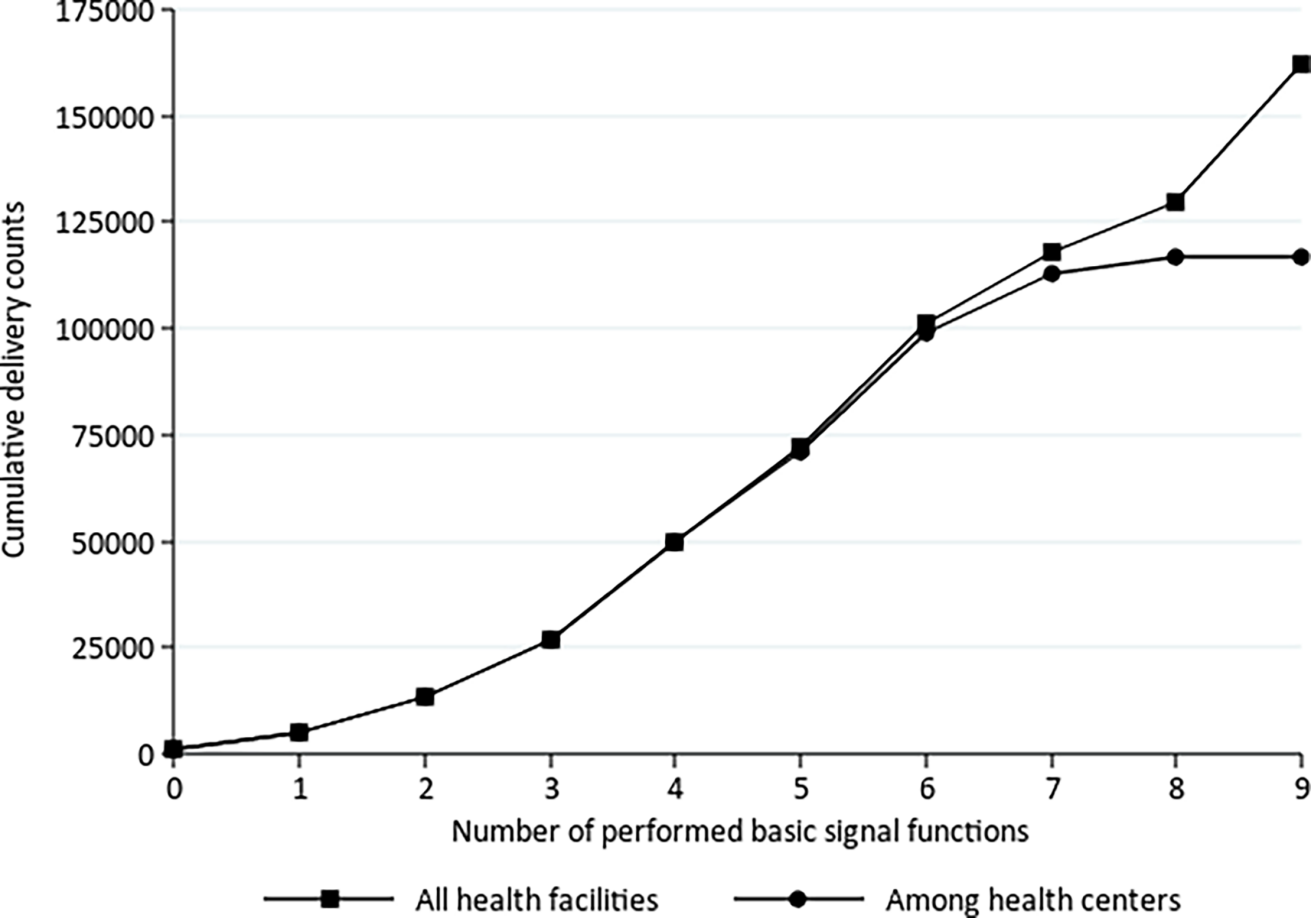
Cumulative number of deliveries, by number of basic signal functions performed.

Researchers and program managers have called for guidance on how the volume of deliveries or “birth load” might assist health system planners to prioritize which facilities should be designated to provide B-EmOC (10). Training and equipping *all* health centers and posts to be fully functioning B-EmOC facilities may not be practical if sites attend very few deliveries; keeping health workers’ skills current and self-confidence high could be difficult for procedures such as manual removal of placenta or removal of retained products when staff attend one delivery or one incomplete abortion a month. Thus, we looked specifically at health centers and posts to explore the relationship between the number of signal functions performed in the last 3 months and the average and median number of deliveries the facilities attended per month. Not surprisingly, we found a positive relationship between the two–as the number of deliveries increased, so did the cumulative number of signal functions performed (Table 4 and Fig 2). The table suggests that in Mozambique a median of 76 or mean of 91 deliveries a month was the threshold for performing all seven basic signal functions. This could be different in other countries.

**Table 4:** Mean, median and range of deliveries per month among health centers and posts, by number of signal functions performed in the 3 months prior to the survey in 2012.

		Health centers & posts		
Number of signal functions performed in last 3 months	Number of health centers & posts	Mean number of deliveries	Median number of deliveries	Range of deliveries
0	16	29	29	0–186
1	66	20	15	0–328
2	117	25	19	0–338
3	152	30	25	0–402
4	176	46	33	5–1092
5	186	48	37	0–919
6 (AVD was performed)	29	76	59	25–664
6 (AVD was not performed)	118	69	52	0–1808
7 (fully functioning)	31	91	76	23–788

SF = signal function; AVD = assisted vaginal delivery

### EmOC indicators–measuring utilization and quality

The indicators for measuring utilization of services and quality of services showed signs of improvement: the proportion of expected births delivered in facilities, measured by the institutional delivery rate, increased from 57% to 67% (Table 5). However, the percentage of deliveries that took place in health centers and posts did not change: 74% in 2007 and 72% in 2012 (data not shown). Met need for EmOC—the percentage of women expected to develop a major obstetric complication who were treated at a facility–also rose from 20% to 26% during the 5-year interval, but remained far below the recommended 100%. If we look at these two rates restricted to fully functioning EmOC facilities, the rates did not substantially change, suggesting that the increases occurred primarily in partially functioning EmOC facilities. The population-based cesarean delivery rate–an indicator that assesses whether women who need life-saving interventions receive them—increased from 2.2% to 2.8%.

**Table 5:** EmOC indicators of utilization, quality and the need for services other than EmOC, by year.

Indicators	2007	2012
Institutional delivery rate		
All facilities	57%	67%
EmOC facilities	17%	19%
Met need for EmOC		
All facilities	20%	26%
EmOC facilities	11%	12%
Cesarean delivery rate	2.2%	2.8%
Direct obstetric case fatality rate	5.2%	2.8%
Institutional stillbirth rate	29.3/1,000 births	19.3/1,000 births
Pre-discharge early neonatal death rate	5.9/1,000 live births	2.2/1,000 live births
Proportion of maternal deaths due to indirect causes	23%	50%

A proxy for quality of care, the aggregated direct obstetric case fatality rate decreased by almost half, from 5.2% to 2.8%, in other words, the rate of dying from a major direct obstetric complication once a woman arrived at a facility decreased by almost half.

Newborn outcomes also improved by 2012: the stillbirth rate decreased by one third (29.3/1,000 births to 19.3) and the pre-discharge early neonatal death rate decreased by two-thirds (5.9/1,000 live births to 2.2). A change in how the data were collected between 2007 and 2012 may also have affected the reduction in early neonatal mortality. In 2007, early neonatal deaths were counted if they occurred within the first 24 hours after birth, while in 2012 they were limited to the first 12 hours. In both cases, deaths occurred before discharge.

Finally, in 2012 half of maternal deaths were due to indirect causes, pointing to an unmet need for services other than EmOC; this proportion consisted primarily of maternal deaths due to malaria, HIV and/or anemia. This change in the distribution of the causes of maternal death has been described elsewhere [28].

#### Maternal cause-specific case fatality rates

The cause-specific case fatality rates (CFRs) for major direct causes of maternal deaths decreased or, in the case of obstructed labor, did not change (1.6% vs. 1.7%) (Table 6). Notably, the CFRs for pre-eclampsia/eclampsia, postpartum hemorrhage (PPH), and ruptured uterus decreased sharply by about two-thirds. The CFRs for ectopic pregnancy and antepartum hemorrhage also indicated reductions of more than 50%. The smallest percentage changes among the CFRs were for women who died of sepsis (17% decrease) and from complications of abortion (20% decrease). The three most lethal causes of maternal mortality in 2007 were uterine rupture, PPH and sepsis, in that order; in 2012, they were the same afflictions but the order differed: sepsis, uterine rupture and PPH. Nevertheless, PPH and pre-eclampsia and eclampsia claimed the largest number of direct maternal deaths in each assessment.

**Table 6:** Direct cause-specific case fatality rates (CFR), by year.

	2007 ^a^ (12 months)			2012 (3 months)			% change^c^
	Events	Deaths	CFR (%)	Events	Deaths	CFR (%)	
Direct causes							
Pre-eclampsia/eclampsia	3,509	246	7.0	1,350	30	2.2	-69%
Postpartum hemorrhage ^b^	1,883	179	9.5	1,137	36	3.2	-66%
Ruptured uterus	943	140	14.8	418	22	5.2	-65%
Ectopic pregnancy	2,263	120	5.3	355	9	2.5	-53%
Antepartum hemorrhage	3,052	137	4.5	919	19	2.1	-53%
Complications of abortion	3,192	112	3.5	251	7	2.8	-20%
Postpartum sepsis	1,441	121	8.4	399	28	7.0	-17%
Obstructed labor	3,334	52	1.6	1,269	21	1.7	+6%

^a^ For this table the weighted numbers are presented for 2007, while in 2012, the absolute numbers collected over the 3-month period are shown.

^b^ Includes retained placenta

^c^ Computed as (CFR_2012_ –CFR_2007_)/CFR_2007_

## Discussion

From 2007 to 2012 important progress in the performance of many of the EmOC signal functions was achieved. However, this progress was not reflected by the indicator on availability of EmOC facilities that complied with the standard but rigid definition of having provided all the signal functions in the three months before the assessment. Availability of fully functioning EmOC facilities decreased largely due to lower reported usage of assisted vaginal delivery by both hospitals and health centers. If AVD were not a requisite for a facility to be fully functioning as B-EmOC, 124 additional facilities would have been classified as B-EmOC (6 hospitals and 118 health centers/posts). Posed differently, if those 124 facilities had performed AVD, the health system would have fulfilled 80% of the minimum recommended number of EmOC facilities, rather than just 28% of the recommended number. Other countries have shown a similar finding, though perhaps not this dramatic [9, 29, 30].

This highly restrictive interpretation of what constitutes an EmOC facility, as this paper points out, fails to reveal where the gaps are. A more in-depth analysis into what signal functions were missing, at what level of facility, and how many were performed at two points in time leads to a more informed discussion for program implementation and policy. Thus, despite the reduced number of EmOC facilities as defined by the “standard” definition of EmOC, this analysis also shows the progress achieved at increasing the provision of life-saving procedures, especially at the health center/post level.

Many reasons could have contributed to the decrease in the reported use of assisted vaginal delivery, but a recent analysis of the readiness to perform the EmOC signal functions in Mozambique suggests that it was not due to a decrease in the availability of equipment [31] and training in B-EmOC in Mozambique continues to include vacuum extraction. To guide practice and policy in Mozambique as well as in other sub-Saharan countries, investigation into why AVD is falling out of favor in some settings but not in others should be of interest to professional societies and the research community. Some voices in the global community have expressed concern for the trauma experienced by the newborn as a result of AVD although researchers calling for caution in its use also support the practice [32]. However, growing evidence from a busy university hospital in Uganda recently showed that intrapartum stillbirths and uterine ruptures significantly declined after re-introducing vacuum extraction into their practice [33]. The low use of AVD has been noted in other sub-Saharan African countries and yet, in neighboring Malawi, its use has steadily increased [34]. The de-skilling of midwives in Mozambique is unfortunate as both assessments showed that many facilities were staffed with midwives able to provide AVD [31].

The modest increases in met need for EmOC and the cesarean delivery rate suggest that more women who needed emergency services received them, but both indicators remain low compared to global benchmarks. Considering that in 2012 67% of expected births took place in facilities, we would expect met need for EmOC to exceed 26%. If 15% of births are expected to develop serious complications that require clinical management and treatment [6], and if those complications were evenly distributed among the 434,508 women who delivered in a facility according to the 2012 assessment (and one might argue that women with complications might disproportionately seek delivery services at a facility), then met need for EmOC would have been at least 58%. Theoretically, low met need for EmOC could be driven by weak diagnostic skills and/or under-recording of obstetric complications. The latter argues for the incorporation of the number of obstetric complications treated into routine health management information systems to reinforce the importance of documenting maternal morbidity. The implications of incomplete recording of complications spill over to the accuracy of the case fatality rates. We must also consider that the denominator for met need, traditionally based on 15% of expected births, is inaccurate, despite numerous studies that support its use [35–38]. These studies as well as the analysis of global met need by countries’ economic status suggest that there may be fewer problems with the 15% and greater challenges with data collection [15].

The cesarean delivery rate remains under 5% indicating that the unnecessary use of cesarean is not likely to be widespread, and yet, a recent study using Mozambique DHS survey data showed underuse of cesarean-sections, especially among socially vulnerable groups in poor-resource regions, and that there might be some overuse among better-off groups [39].

The cause-specific case fatality rates continue high like other high burden maternal mortality countries in sub-Saharan Africa [5], but the decreases also suggest that health workers achieved some success at preventing maternal deaths from major direct causes except for obstructed labor. Whether improvements can be traced to better diagnostic skills and/or improved clinical treatment of complications, or data quality problems (e.g. increased under-reporting of complications that would increase the denominators for the CFRs) is not clear. Despite the large improvements in the CFRs, efforts are needed to support health workers as they continue to tackle what are still unacceptably high rates.

The doubling of the proportion of maternal deaths due to indirect causes (from 23% to 50%) should serve as a call for primary health care facilities and agents to play an even more aggressive role in preventing and treating malaria, HIV and anemia during pregnancy. Indeed, during the 5-year interval the Ministry of Health expanded the availability of primary health care by opening more health facilities and training more health workers. According to a 2015 household survey, more than half the population of pregnant women had attended four or more antenatal visits, two-thirds of homes had one or more insecticide treated bednet for malaria protection, and more than half of the women who gave birth in the last two years had had at least one dose of sulfadoxine pyrimethamine (fansidar) as intermittent preventive treatment [40]. It is also clear that the earlier an HIV pregnant woman begins taking antiretrovirals, the lower the risk of death during pregnancy and childbirth [41]. Platforms for preventive services exist but coverage could be higher.

While efforts in Mozambique to strengthen the primary health care facilities have shown progress, most institutional maternal and newborn deaths take place in hospitals, many of whom are referrals [42], but as we saw in this study, only 51% of hospitals provided all 9 signal functions. In the 2012 EmONC assessment, two-thirds of maternal deaths took place in hospitals suggesting that women with complications actively sought or were referred by the primary health care level to more comprehensive levels of care for treatment. Not only must delays in referral be minimized but all hospitals, assuming they have the infrastructure or the potential for infrastructure, should be supported to perform all signal functions, including blood transfusions and obstetric surgery, so that they can fulfill their role as referral centers.

A strength of this study was the opportunity to use two national facility-based assessments with similar methodologies that allowed comparisons of critical elements of the health system that are not collected in other national surveys. Although methodologies were similar, small differences in questionnaire design led to several limitations. First, the service statistics of 2012 were collected for only one 3-month period and therefore did not reflect seasonal variations in access to care, which might have affected the utilization of services such as institutional delivery or the distribution of maternal mortality [43]. On the other hand, the extraction of these events from registers is tedious and it is possible that a 3-month reference period resulted in less data collection fatigue. Second, as noted above, differences between the two questionnaires made the comparison of cause of maternal death distributions a challenge. In 2007 the questionnaire did not include a category of unknown/unspecified deaths, and instead a very large number of “other direct” maternal deaths were reported. Also, indirect causes of death were categorized differently in 2012 by combining co-morbidities, making cause-specific comparisons difficult. Finally, it is not clear how women who died but had evidence of multiple complications were ultimately classified, and this might have differed across assessments. The standard instruction for the data collector was to choose the direct cause over the indirect cause. Finally, the 12-hour (2012 assessment) or 24-hour (2007 assessment) reference period for early neonatal deaths may have contributed to a lower pre-discharge neonatal mortality rate in 2012.

## Conclusions

Numerous signs of improvement were observed in this 5-year period, which bode well for maternal and newborn indicators in the future. Women appear to widely utilize facility-based intrapartum care but the quality of that care, at least for direct obstetric complications, should remain a high priority, as should further emphasis on preventing and controlling deaths due to indirect causes.

The indicator of EmOC service availability, which is often used for planning EmONC networks, should be explored fully as we have done in this paper. In this case, the decline in the use of assisted vaginal delivery explained much of the difference between the two years, masking improvements in more frequent utilization and performance of other life-saving procedures and treatment of women and newborns.
